# Increasing proportion of vancomycin-resistance among enterococcal bacteraemias in Switzerland: a 6-year nation-wide surveillance, 2013 to 2018

**DOI:** 10.2807/1560-7917.ES.2020.25.35.1900575

**Published:** 2020-09-03

**Authors:** Vanja Piezzi, Michael Gasser, Andrew Atkinson, Andreas Kronenberg, Danielle Vuichard-Gysin, Stephan Harbarth, Jonas Marschall, Niccolò Buetti, on behalf of the Swiss Centre for Antibiotic Resistance (ANRESIS), on behalf of the National Centre for Infection Control (Swissnoso)

**Affiliations:** 1 Department of Infectious Diseases, Bern University Hospital, University of Bern, Bern, Switzerland; 2 Institute for Infectious Diseases, University of Bern, Bern, Switzerland; 3 Department of Internal Medicine, Cantonal Hospital Muensterlingen, Thurgau Hospital Group, Kreuzlingen, Switzerland; 4 Infection Control Programme, University of Geneva Hospitals and Faculty of Medicine, Geneva, Switzerland; 5 IAME, DeSCID team, INSERM, Université Paris Diderot and Sorbonne Paris Cité, Paris, France; 6 The members of the advisory board of ANRESIS are acknowledged at the end of the article; 7 The members of Swissnoso are acknowledged at the end of the article

**Keywords:** bacteraemia, Enterococci, Vancomycin resistant, VRE, surveillance, epidemiology

## Abstract

**Background:**

Vancomycin-resistant enterococci (VRE), mostly *Enterococcus faecium*, are multidrug-resistant microorganisms that can cause nosocomial infections. VRE has increased throughout many European countries, but data from Switzerland are scarce.

**Aim:**

The aim of this work was to characterise the epidemiology of enterococcal bacteraemias in Switzerland with a focus on VRE.

**Methods:**

In this observational study, we retrospectively investigated bacteraemias from 81 healthcare institutions from January 2013 to December 2018 using data from the Swiss Centre for Antibiotic Resistance. Only the first blood isolate with *E. faecalis* or *E. faecium* from an individual patient was considered. We analysed the annual incidences of enterococcal bacteraemias and determined the proportion of VRE over time. We also assessed epidemiological factors potentially associated with VRE bacteraemia.

**Results:**

We identified 5,369 enterococcal bacteraemias, of which 3,196 (59.5%) were due to *E. faecalis* and 2,173 (40.5%) to *E. faecium*. The incidence of enterococcal bacteraemias increased by 3.2% per year (95% confidential interval (CI): 1.6–4.8%), predominantly due to a substantial increase in *E. faecalis* bacteraemic episodes. Vancomycin resistance affected 30 (1.4%) *E. faecium* and one *E. faecalis* bacteraemic episodes. Among all *E. faecium* bacteraemias, the proportion of vancomycin-resistant isolates increased steadily from 2013 to 2018 (2% per year; 95% CI: 1.5–2.9%). No independent epidemiological factor for higher prevalence of vancomycin-resistant *E. faecium* bacteraemias was identified.

**Conclusions:**

Vancomycin-resistant *E. faecium* bacteraemias remain infrequent in Switzerland. However, an important increase was observed between 2013 and 2018, highlighting the need for implementing active surveillance and targeted prevention strategies in the country.

## Introduction

*Enterococcus faecalis* and *E. faecium* are the most common enterococci in the human gastrointestinal flora and the two most common species responsible for invasive enterococcal infections [1]. In Europe, *E. faecalis* and *E. faecium* are the fourth and fifth most frequent causative pathogen of bloodstream infections and a significant increase in enterococcal bacteraemias, particularly *E. faecium*, has been observed from 2002 to 2008 [2]. A similar trend was reported up to 2014 in a Swiss surveillance study [3].

In the last decades, vancomycin-resistant enterococci (VRE), mostly *E. faecium*, emerged worldwide as nosocomial multidrug-resistant microorganisms [4-6]. The morbidity and mortality associated with VRE bacteraemia are increased compared with infections due to susceptible enterococci, despite the use of effective antibiotics [7]. In Europe (countries in the European Union and in the European Economic Areas), according to the 2018 surveillance report of the European Antimicrobial Resistance Network (EARS-Net), the national percentages for vancomycin resistance among *E. faecium* isolates ranged from 0.0% to 59.1%. Several countries with already high resistance rates reported further increases during 2014–2018 [8]. Switzerland is not part of the EARS-Net and national vancomycin-resistant *E. faecium* data have not been published for infection control purposes to date. However, since 2009 several small VRE outbreaks have been documented in the country [9-13]. At the beginning of 2018, a large monoclonal outbreak with vancomycin-resistant *E. faecium* sequence type (ST) 796 affected several hospitals in the north-eastern part of Switzerland [14].

We hypothesised that the national epidemiological situation of enterococcal bacteraemias is evolving quickly and that invasive vancomycin-resistant *E. faecium* infections are increasing. We therefore wanted to describe patterns and trends of enterococcal bacteraemias in Switzerland from 2013 to 2018 with a focus on vancomycin-resistant isolates, using data from the Swiss Centre for Antibiotic Resistance (ANRESIS).

## Methods

### Study design and setting

We performed a retrospective observational analysis of enterococcal bacteraemias in Switzerland over 6 years, from 1 January 2013 to 31 December 2018. We used prospectively collected epidemiological and microbiological data from a nationwide microbiology laboratory surveillance system.

### Data source

The isolates were identified by a search in the ANRESIS database for all blood cultures positive for *E. faecalis* or *E. faecium*. ANRESIS collects all routine antibiotic resistance data from currently 24 clinical microbiology laboratories homogeneously distributed across Switzerland. Each participating laboratory gathers data from several hospitals and submits results on a voluntary basis (weekly or monthly) to a central database located at the Institute for Infectious Diseases, University of Bern, Switzerland. We restricted the dataset to hospitals that continuously reported bacteraemias during the whole study period (hospitals not reporting bacteraemias in 2013 were included if they reported bacteraemias in ≥ 4 calendar years from 2014 to 2018). The information on hospital size and patient days was obtained using national data on hospital statistics [15].

### Isolates included, microbiological and epidemiological data

Positive blood cultures were interpreted as bacteraemic episodes (i.e. bacteraemias). For this study, only bacteraemic episodes with *E. faecalis* and *E. faecium* were included. We excluded isolates of other enterococcal species (*e.g. E. gallinarum* or *E. casseliflavus*). Only the first isolate from an individual patient was considered for the current analysis.

The microbiological data included enterococcal species and antibiotic susceptibility patterns. Antimicrobial susceptibility was tested in the participating laboratories according to the European Committee on Antimicrobial Susceptibility Testing (EUCAST) guidelines (https://eucast.org/) or the Clinical and Laboratory Standards Institute (CSLI) guidelines in a single laboratory (https://clsi.org/). Resistant isolates were defined as those that were resistant or intermediately susceptible to vancomycin, regardless of the underlying mechanism.

The epidemiological data available for each isolate allowed the stratification by sex and age, hospital type (community hospitals vs university hospitals), hospital size (< 200, 200–400, > 400 beds), hospital departments (intensive care unit (ICU) vs non-ICU departments) and geographical region (north-east vs. south-west). Clinical data (e.g. clinical diagnosis, source of infection, therapy, length of the hospital stay and outcomes) were not available.

### Statistical analysis

Characteristics of patients with bacteraemia were described as count (per cent) or median (interquartile range (IQR)) for qualitative and quantitative variables, respectively, and were compared between groups using chi-squared or Wilcoxon test, as appropriate. The incidence of bacteraemias was calculated as the number of positive blood samples per 100,000 patient days. Incidence trends were assessed using Poisson regression with the offset being the log of patient days. The prevalence of vancomycin resistance among *E. faecium* was calculated as the number of resistant strains over the total number of *E. faecium* isolates. Changes in the percentage were assessed using the Cochrane–Armitage test and univariate logistic models.

We performed additional explanatory comparative analysis in order to identify variables associated with VRE.

Confidential intervals (CI) were calculated and p values < 0.05 were considered to be statistically significant. We performed all analyses using R (Version 3.5.1). The current study followed the Strengthening the Reporting of Observational Studies in Epidemiology (STROBE) guidelines for observational studies [16].

### Validation of database

We verified the accuracy of the ANRESIS database in reporting antibiotic resistance. We compared ANRESIS data with the results of a recently published nationwide survey on VRE epidemiology in Swiss hospitals performed in collaboration with the National Centre for Infection Control (Swissnoso) [12]. This survey included high-quality VRE data on samples obtained from patients with bacteraemic episodes, invasive infections and screening, which were collected by individual infection prevention specialists in 142 healthcare institutions in Switzerland. The numbers of VRE bacteraemias of individual hospitals, gathered from 1 January 2015 to 31 March 2018 from 55 healthcare institutions, each represented both in the ANRESIS and in the survey database, were compared to test the accuracy of the ANRESIS database. This analysis was performed using Wilcoxon signed rank tests for paired data.

### Ethical statement

As the analysis was performed on anonymised non-genetic surveillance data, ethical consent was not required according to the Swiss law for research on humans (Article 33, Paragraph 2, Human Research Act).

## Results

### Isolates and hospitals included in the study

**Figure 1:**
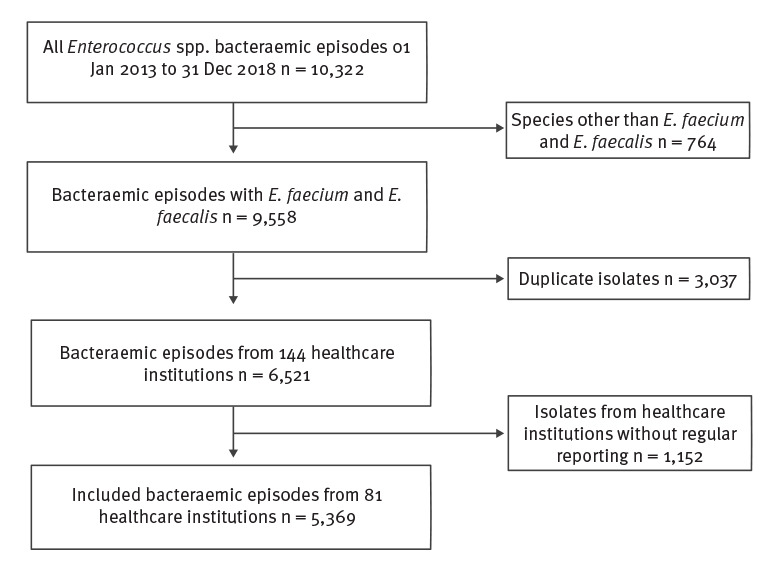
Selection of enterococcal bacteraemic episodes for the nationwide surveillance study on vancomycin-resistance among enterococcal bacteraemias, Switzerland, 2013–2018 (n = 10,322 bacteraemic episodes)

**Figure 2:**
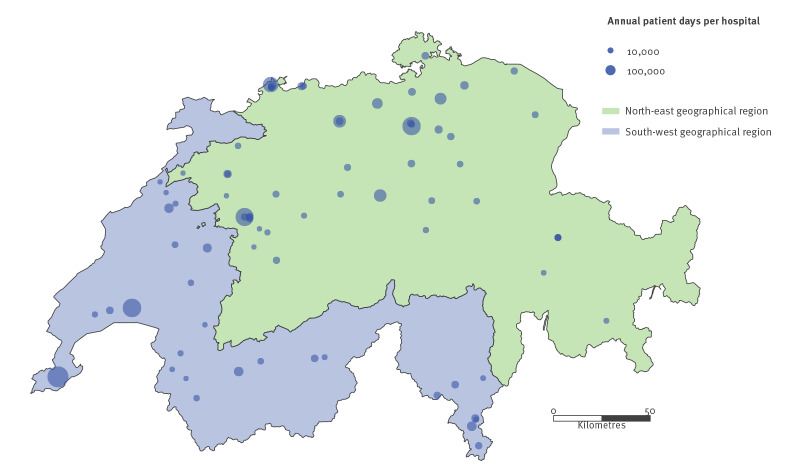
Swiss healthcare institutions included in the nationwide surveillance study on vancomycin resistance among enterococcal bacteraemias, 2013–2018 (n = 81 institutions)

From 1 January 2013 to 31 December 2018, data on 6,521 bacteraemic episodes with *E. faecium* and *E. faecalis* from 144 Swiss healthcare institutions were submitted to ANRESIS. Sixty-three healthcare institutions were excluded because the collaboration with ANRESIS started after 2013 or the data reporting was incomplete. We included a total of 5,369 enterococcal bacteraemic episodes (Figure 1). The selected 81 healthcare institutions are displayed in Figure 2 and represented ca 44% of all patient days in Switzerland during the whole study period. All five Swiss university hospitals were included in the analysis (a detailed comparison between included and excluded isolates is available in the Supplementary Table S1).

*E. faecalis*: *Enterococcus faecalis*; *E. faecium*: *Enterococcus faecium*.

The disk sizes are proportional to the hospitals’ annual patient days. The background colours on the map represent geographical regions of Switzerland (green: north-east; blue: south-west).

### Epidemiological and microbiological characteristics of bacteraemia-derived isolates

Of the 5,369 samples obtained from bacteraemic patients, 2,173 (40.5%) were due to *E. faecium* and 3,196 (59.5%) to *E. faecalis*. Epidemiological characteristics of patients with bacteraemia are summarised in Table 1. Enterococcal bacteraemias occurred more frequently in men (n = 3,614; 67.3%) and the median age of all patients was 70 years (IQR: 60–80). The majority of the samples (2,760; 51.4%) were observed in hospitals with > 400 beds and 2,408 (44.9%) were from university hospitals. Almost 15 per cent of the patients (n = 780) were hospitalised in the ICU at the time of blood sampling.

**Table 1:** Epidemiological characteristics of patients with *Enterococcus faecium* and *E. faecalis* bacteraemia, Switzerland, 2013–2018 (n = 5,369 bacteraemias)

Characteristics		All bacteraemias (n = 5,369)	*E. faecium* (n = 2,173)	*E. faecalis* (n = 3,196)	p value
Age – year, median (IQR)		70 (60–80)	70 (55–75)	70 (60–80)	< 0.001
Male sex – n (%)		3,614 (67.3)	1,388 (63.9)	2,226 (69.6)	< 0.001
Department – n (%)	ICU	780 (14.5)	420 (19.3)	360 (11.3)	< 0.001
	Non-ICU	4,589 (85.5)	1,753 (80.7)	2,836 (88.7)	
Hospital type – n (%)	University hospital	2,408 (44.9)	1,177 (54.2)	1,231 (38.5)	< 0.001
	Community hospital	2,961 (55.1)	996 (45.8)	1,965 (61.5)	
Hospital size – n (%)	> 400 beds	2,760 (51.4)	1,276 (58.7)	1,484 (46.4)	< 0.001
	200–400 beds	1,321 (24.6)	525 (24.2)	796 (24.9)	
	< 200 beds	1,288 (24.0)	372 (17.1)	916 (28.7)	
Geographical region – n (%)	South-west	2,066 (38.5)	814 (37.5)	1,252 (39.2)	0.22
	North-east	3,303 (61.5)	1,359 (62.5)	1,944 (60.8)	
Year – n (%)	2013	762 (14.2)	326 (15.0)	436 (13.6)	cf trend analysis^a^
	2014	860 (16.0)	333 (15.3)	527 (16.5)	
	2015	948 (17.7)	402 (18.5)	546 (17.1)	
	2016	909 (16.9)	361 (16.6)	548 (17.1)	
	2017	968 (18.0)	391 (18.0)	577 (18.1)	
	2018	922 (17.2)	360 (16.6)	562 (17.6)	
Vancomycin resistance – n (%)		31 (0.6)	30 (1.4)	1 (0)	< 0.001

ICU: intensive care unit; IQR: interquartile range.

^a^ For detailed analysis, see trend data.

Epidemiological characteristics differed between individuals with *E. faecium* and *E. faecalis* bacteraemias. *E. faecalis* bacteraemias were more frequently observed in community hospitals compared to *E. faecium* bacteraemias (61.5% vs 45.8% respectively; p < 0.001) and in smaller hospitals (28.7% vs 17.1%; p < 0.001). Patients with *E. faecium* were more likely to be hospitalised in the ICU at time of bacteraemia diagnosis than patients with *E. faecalis* (19.3% vs 11.3%; p < 0.001). Antimicrobial resistance against vancomycin was generally low (n = 31; 0.6%) and was mainly observed among *E. faecium* isolates (1.4% vs 0% of *E. faecalis* bacteraemias; p < 0.001).

### Trends in annual incidences of enterococcal bacteraemias between 2013 and 2018

**Figure 3:**
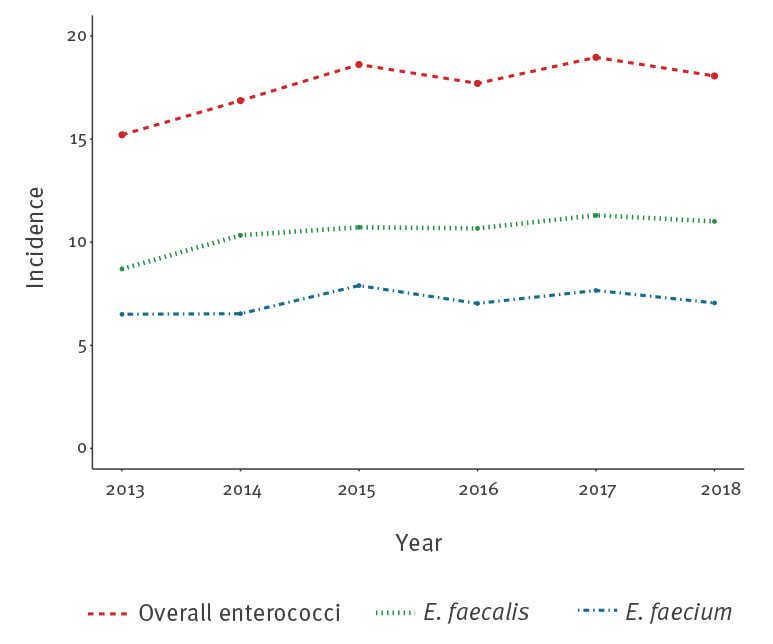
Time trends for annual incidences of bacteraemias (number of bacteraemias per 100,000 patient days) due to *Enterococcus faecalis* and *E.* *faecium*, Switzerland, 2013–2018 (n = 5,369 bacteraemias)

The time trends for the annual incidences of enterococcal bacteraemias are shown in Figure 3. The total number of episodes reported increased from 762 (2013) to 922 (2018), which corresponded to an upward trend in incidence from 15.2 to 18.1 per 100,000 patient days (p < 0.01). We observed a 3.2% increase per year (95% CI: 1.6–4.8%; p < 0.001) that was explained by an increasing incidence of *E. faecalis* bacteraemias from 8.7 to 11.0 per 100,000 patient days (mean: 3.9% per year; 95% CI: 1.9–6.0%; p < 0.001). There was no significant increase in *E. faecium* incidence over the study period (from 6.5 to 7.1 per 100,000 patient days; p = 0.10).

The term ‘overall enterococci’ refers to the species included in this study.

In a post hoc subgroup analysis of *E. faecalis*, we found a marginally significant increase in non-ICU departments (from 86.7% in 2013 to 90.6% in 2018; p = 0.04, data not shown), whereas no significant trend was observed among other subgroups (i.e. age, sex, region, hospital size and hospital type).

### Vancomycin-resistant isolates with time trend for *Enterococcus faecium* prevalence

**Figure 4:**
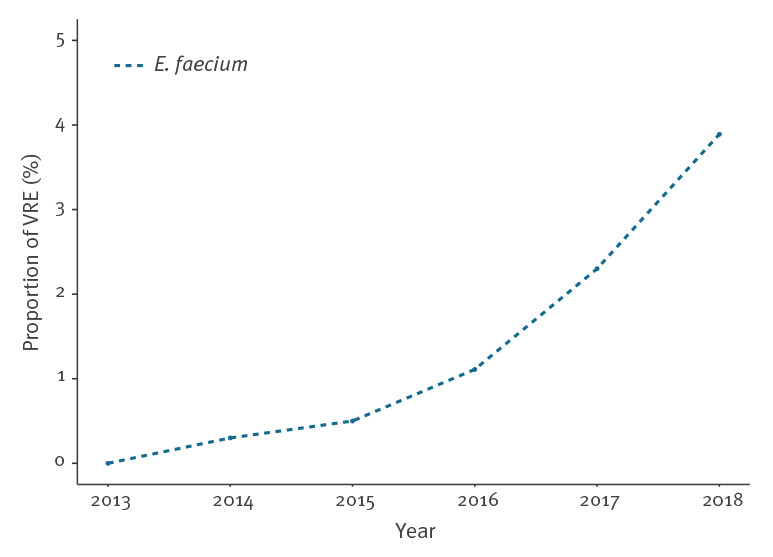
Time trend for the proportion of vancomycin resistance among *Enterococcus faecium* bacteraemias, Switzerland, 2013–2018 (n = 2,173 *E. faecium* bacteraemias)

A total of 30 vancomycin-resistant *E. faecium* isolates were identified over the study period*.* Their proportion increased from 0% (n = 0/326) in 2013 to 3.9% (n = 14/360) in 2018, corresponding to a 2.0% annual increase (95% CI: 1.5–2.9; p < 0.01) (Figure 4). Only one bacteraemic isolate of *E. faecalis*, sampled in 2015, was resistant to vancomycin.

VRE: vancomycin resistance among *E. faecium* bacteraemias*.*

#### Additional analyses

During the first 3 years of the study period, we encountered only three blood cultures positive for vancomycin-resistant *E. faecium*, whereas between 2016 and 2018 we observed 27. To identify epidemiological factors associated with the upward trend of VRE bacteraemias, we performed a post hoc analysis only for 2016–2018, which revealed no significant factor associated with the detection of vancomycin-resistant *E. faecium* (Table 2).

**Table 2:** Difference between epidemiological characteristics of vancomycin-susceptible and vancomycin-resistant *Enterococcus faecium* bacteraemias, Switzerland, 2016–2018 (n = 1,112 bacteraemias)

Characteristic		VSE bacteraemia (n = 1,085)	VRE bacteraemia (n = 27)	p value
Age – year, median (IQR)		70 (55–75)	65 (55–75)	0.60
Male sex – n (%)		685 (63.1)	15 (55.6)	0.55
Department – n (%)	ICU	210 (19.4)	7 (25.9)	0.55
	Non-ICU	875 (80.6)	20 (74.1)	
Hospital – n (%)	University hospital	573 (52.8)	17 (63.0)	0.40
	Community hospital	512 (47.2)	10 (37.0)	
Hospital size – n (%)	> 400 beds	619 (57.1)	19 (70.4)	0.27
	200–400 beds	267 (24.6)	6 (22.2)	
	< 200 beds	199 (18.3)	2 (7.4)	
Geographic region – n (%)	South-west	420 (38.7)	9 (33.3)	0.71
	North-east	665 (61.3)	18 (66.7)	
Year – n (%)	2016	357 (32.9)	4 (14.8)	cf trend analysis^a^
	2017	382 (35.2)	9 (33.3)	
	2018	346 (31.9)	14 (51.9)	

ICU: intensive care unit; IQR: interquartile range; VRE: vancomycin resistant *Enterococcus faecium*; VSE: vancomycin susceptible *Enterococcus faecium*.

^a^ For detailed analysis, see trend data.

### Validation of the database used

Among the 55 healthcare institutions included in the accuracy analysis, 23 and 27 VRE bacteraemias were observed in the ANRESIS and in the survey database, respectively. Regarding the accuracy analysis (Supplementary Figure S2), no significant difference in the reported number of VRE episodes of individual hospitals between the two databases was found in a Wilcoxon signed rank tests for paired data (p = 0.28). Two hospitals (3.6%) reported an identical number of positive samples and from 41 hospitals (74.6%) no bacteraemic episodes with VRE were detected in either database. Nine institutions (16.4%) showed a discrepancy of one sample, three (5.5%) a discrepancy of more than one sample. In summary, a high congruency for bacteraemias was observed between the two databases.

## Discussion

In this work, we report annual incidences of enterococcal and VRE bacteraemias in Switzerland and their evolution over a recent 6-year period, using data from ANRESIS. Including more than 80 healthcare institutions and representing almost half of all patient days accrued in Switzerland per year, this database can be considered as representative for the entire country. As Switzerland does not participate in the EARS-Net, our data provide essential information on the epidemiology of invasive enterococcal infections in Europe. Moreover, given the geographical location of the country at the crossroads of Europe, the findings are useful for benchmarking purposes with other European countries.

From January 2013 to December 2018, a total of 5,369 bacteraemic episodes with *E. faecalis* and *E. faecium* were characterised. *E. faecalis* was the most prevalent *Enterococcus* species in all geographical regions of Switzerland. This was consistent with the results of the global antimicrobial surveillance programme SENTRY (1997–2016), which found a prevalence of enterococcal bloodstream infections reaching 10.7% of all bacteraemic isolates in North America and 8.1% in Europe, respectively [6].

In line with European trends [2,17], an increase in annual enterococcal bacteraemia incidence was found in Switzerland. This was mainly caused by an increase in *E. faecalis* bacteraemic episodes, particularly in non-ICU hospital departments. The rate of *E. faecalis* causing bacteraemias in Europe, however, remained stable during the last decade [17]. We do not have enough information to explain the persisting upward trend for *E. faecalis* in non-ICU departments in Switzerland. Further studies should focus on this setting in order to better delineate these findings.

Our study constitutes the first report on trends of VRE bacteraemia in Switzerland and an emerging proportion of vancomycin-resistant *E. faecium,* up to 3.9% in 2018, was observed. In our surveillance, we nevertheless found no significant increase in *E. faecium* incidence. In contrast to previously published data, which demonstrated the additional effect of antibiotic-resistant bacteria on the total burden of nosocomial bloodstream infections [18], the trends encountered here suggest that VRE *E. faecium* are replacing susceptible *E. faecium* isolates.

A global increase in the rates of vancomycin resistance among *E. faecium* was observed over the last few decades: according to surveillance data until 2016, this reached 21.0% in the United States and 9.9% in Europe [6]. Among European countries, however, a recent EARS-Net report described significant variability in vancomycin resistance rates for *E. faecium* (from 0% in Iceland, Luxemburg and Slovenia to 59.1% in Cyprus in 2018) [8]. Concerning the VRE proportion in countries neighbouring Switzerland, France and Austria exhibited low prevalences (0.6% and 2.1%, respectively) and decreasing trends until 2018 [8]. In Germany, in contrast, the VRE proportion among enterococcal bloodstream infection increased dramatically in the last years [19,20], with the proportion in 2018 being as high as 23.8% [8]. In Italy, an increasing VRE incidence was recently reported as well [8]. The exact reasons for the heterogeneous geographical VRE distribution pattern are currently unknown, but are probably linked to infection control and antibiotic usage practices [21].

Since 2018, due to a large outbreak with an emergent vancomycin-resistant clone (ST796) in the north-east region of Switzerland [14], the awareness of this nosocomial pathogen has risen sharply. We describe a significant increase of the proportion of vancomycin-resistant isolates among *E. faecium* bacteraemias, with no specific characteristic significantly associated to the higher prevalence of such isolates. Notably, no difference in proportions between the regions was observed. In light of these considerations, we hypothesise that the spread of VRE in Switzerland appears to be homogeneously distributed throughout the country and started before the aforementioned outbreak.

Assuming that the observed trends are real, we wondered whether they could be associated with the hospital setting. The included surveillance data, however, did not provide enough details to identify the role of healthcare-associated bacteraemias. A national survey in 2018 reported an increasing number of VRE outbreaks in Swiss hospitals, thus suggesting nosocomial dissemination of VRE [12]. Moreover, a surveillance study conducted in Norway from 2006 to 2017, a country with low VRE prevalence, reported that over 85% of the VRE cases were associated with hospital outbreaks [22]. Also in Denmark, a cohort study revealed that the majority of enterococcal bacteraemias were hospital-acquired [23].

Several factors may explain the observed trends in VRE bacteraemias. First, several Swiss institutions have not yet established an admission screening policy for detecting VRE carriers and reporting for VRE is not mandatory, which reduces the likelihood of an early detection of VRE clusters [24]. Due to the relatively low risk (4%) of developing a bacteraemia for VRE-colonised patients [25], active surveillance is essential to thoroughly understand VRE epidemiology. Second, harmonised national infection prevention recommendations (e.g. contact precautions, single room, environmental decontamination) have not yet been developed and implemented throughout the country [26]. Third, the consumption of cephalosporins and vancomycin, both important risk factors for VRE acquisition [27,28], increased over the last decade in Swiss hospitals [29]. Fourth, a national survey conducted in 2018 highlighted a substantial heterogeneity regarding the VRE outbreak containment strategies adopted by the different institutions [12]. To cope with this emergent situation, a national VRE task force was created in 2018 in order to improve surveillance and facilitate the early detection of VRE outbreaks, to harmonise the communication between hospitals, and to prepare national guidelines with specific prevention measures [30]. Moreover, a national antimicrobial stewardship programme to decrease antibiotic consumption were recently introduced [26].

There are several limitations to the present study. First, we extrapolated incidence based on the epidemiology of 44% of all Swiss healthcare institutes and, consequently, the generalisability of our findings may be somewhat limited. Nevertheless, in order to better describe our cohort of hospitals, we reported differences between included and excluded hospitals in a subgroup analysis. Second, we cannot exclude the possibility of multiple inclusions of follow-up species from one individual after transfer to a region served by another laboratory. Third, similarly to other resistance surveillance databases, we did not have access to clinical data. For example, we were unable to distinguish between clinical infection and contamination. However, *Enterococcus* spp. is not a typical contaminant in blood cultures and therefore more likely to be the cause of bacteraemia if recovered [31,32]. Fourth, no information on molecular resistance mechanisms was available. Moreover, the clonality between enterococcal strains was not assessed and, therefore, we were unable to confirm or rule out regional or inter-regional VRE spread of specific clones. Finally, due to the low incidence of VRE bacteraemias, we did not include cluster effects for the different healthcare institutions in the statistical analysis and the results of the subgroup analysis may have been underpowered.

In conclusion, we detected an increasing proportion of bacteraemias due to vancomycin-resistant *E. faecium* from 2013 to 2018 in Switzerland. In order to reduce the spread of VRE in Switzerland, both detection strategies and infection prevention and containment measures should be further developed and uniformly implemented.

## Swiss Centre for Antibiotic Resistance (ANRESIS) advisory board

In alphabetical order: A. Burnens, Synlab Suisse, Switzerland; A. Cherkaoui, Bacteriology Laboratory, Geneva University Hospitals, Switzerland; C. Corradi, Federal Office of Public Health, Bern, Switzerland; O. Dubuis, Viollier AG, Basel, Switzerland; A. Egli, Clinical Microbiology, University Hospital Basel, Switzerland; V. Gaia, Department of microbiology, EOLAB, Bellinzona, Switzerland; D. Koch, Federal Office of Public Health, Bern, Switzerland; A. Kronenberg, Institute for Infectious Diseases, University of Bern, Switzerland; S. L. Leib, Institute for Infectious Diseases, University of Bern, Switzerland; P. Nordmann, Molecular and Medical Microbiology, Department of Medicine, University Fribourg, Switzerland; V. Perreten, Institute of Veterinary Bacteriology, University of Bern, Switzerland; J.-C. Piffaretti, Interlifescience, Massagno, Switzerland; G. Prod’hom, Institute of Microbiology, Centre Hospitalier Universitaire Vaudois, Lausanne, Switzerland; J. Schrenzel, Bacteriology Laboratory, Geneva University Hospitals, Geneva, Switzerland; A. F. Widmer, Division of Infectious Diseases and Hospital Epidemiology, University of Basel, Switzerland; G. Zanetti, Service of Hospital Preventive Medicine, Centre Hospitalier Universitaire Vaudois, Lausanne, Switzerland; R. Zbinden, Institute of Medical Microbiology, University of Zürich, Switzerland.

## Swissnoso members

In alphabetical order: Carlo Balmelli, Lugano; Marie-Christine Eisenring, Sion; Stephan Harbarth, Geneva; Stefan P. Kuster, Zurich; Jonas Marschall, Bern; Didier Pittet, Geneva; Christian Ruef, Zurich; Hugo Sax, Zurich; Matthias Schlegel, St. Gallen; Alexander Schweiger, Basel; Laurence Senn, Lausanne; Nicolas Troillet, Sion; Andreas F. Widmer, Basel; Giorgio Zanetti, Lausanne.

## Supplementary Data

52000 Supplementary Material
