# Supplementary material for: Increasing proportion of vancomycin-resistance among enterococcal bacteraemias in Switzerland: a 6-year nation-wide surveillance, 2013 to 2018
Source: Euro Surveill. 2020 Sep 3;25(35):1900575. doi: 10.2807/1560-7917.ES.2020.25.35.1900575 (PMC7472687; doi:10.2807/1560-7917.ES.2020.25.35.1900575)
Supplement: Supplementary Material [file 1900575_Supplement.pdf]

Supplementary material

This supplementary material is hosted by *Eurosurveillance* as supporting information alongside the article **"Increasing proportion of vancomycin-resistance among enterococcal bacteremia in Switzerland: a 6-years nation-wide surveillance, 2013-2018"**, on behalf of the authors, who remain responsible for the accuracy and appropriateness of the content. The same standards for ethics, copyright, attributions and permissions as for the article apply. Supplements are not edited by *Eurosurveillance* and the journal is not responsible for the maintenance of any links or email addresses provided therein.

Supplementary Table S1

Differences in baseline characteristics between included and excluded bacteremias

	**Included (n = 5369)**	**Excluded (n = 1152)**	**p-value**
Male sex – n (%)	3614 (67.3)	651 (56.5)	<0.001
Department – n (%) #			0.02
- ICU	780 (14.5)	67 (5.8)	
- Non-ICU	4589 (85.5)	547 (94.2)	
Hospital type – n (%)			<0.001
- University hospital	2408 (44.9)	0 (0)	
- Community hospital	2961 (55.1)	1152 (100)	
Geographic Region – n (%)			0.30
- Southwest	2066 (38.5)	424 (36.8)	
- Northeast	3303 (61.5)	728 (63.1)	

Footnote: n (%): count (percent); ICU: intensive care unit; # 538 missing data among excluded bacteremias

Supplementary Figure S2

Number of VRE bacteremias reported from individual hospitals

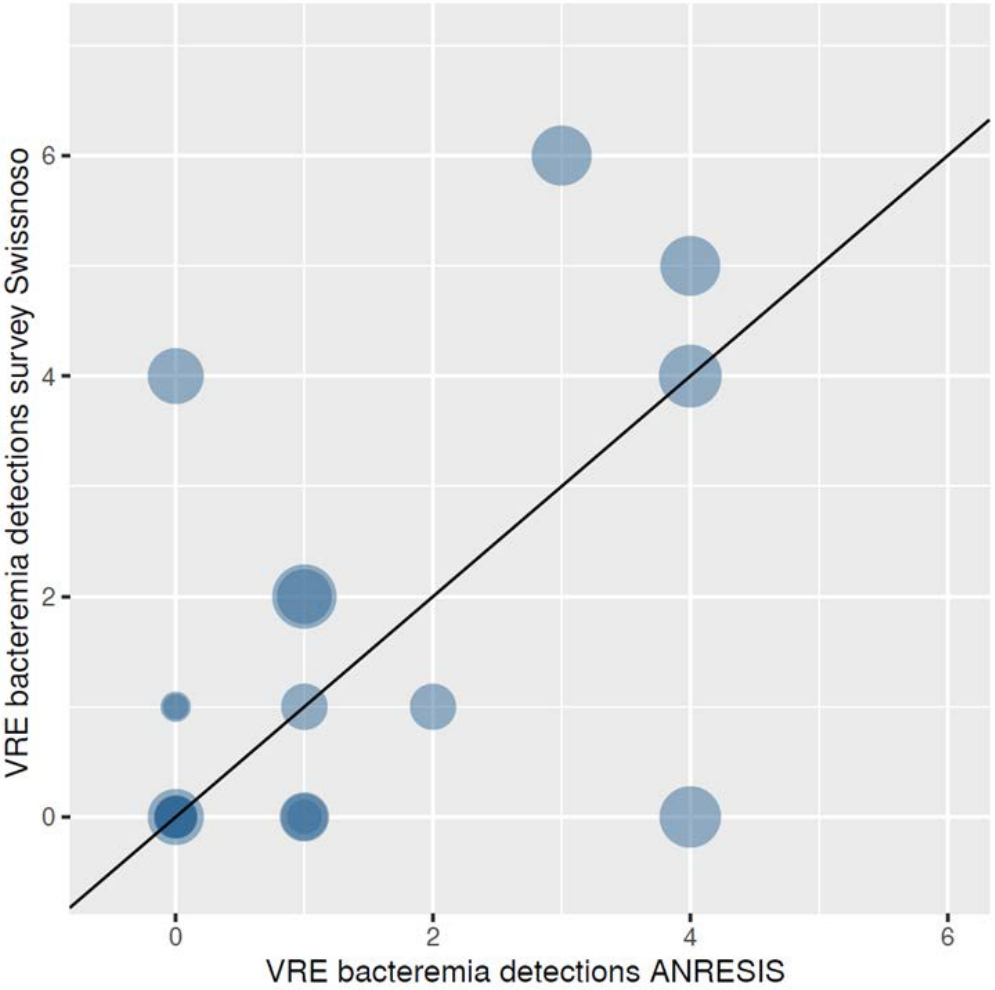

The point sizes are proportional to the hospitals' annual patient days, the straight line represents the x=y function (i.e., same number of samples in both databases).
